# Dietary Capsaicin Exacerbates Gut Microbiota Dysbiosis and Mental Disorders in Type 1 Diabetes Mice

**DOI:** 10.3390/nu17030593

**Published:** 2025-02-06

**Authors:** Xiaohui Zhang, Houjia Hu, Yue Zhang, Shuting Hu, Jiaqin Lu, Weijie Peng, Dan Luo

**Affiliations:** 1 Department of Physiology, School of Basic Medical Sciences, Jiangxi Medical College, Nanchang University, Nanchang 330006, China; 406400220018@email.ncu.edu.cn (X.Z.); huhoujia@tmu.edu.cn (H.H.); 406400230076@email.ncu.edu.cn (Y.Z.); 24520241154778@stu.xmu.edu.cn (J.L.); 2 School of Pharmaceutics, Jiangxi Medical College, Nanchang University, Nanchang 330006, China; 406700220009@email.ncu.edu.cn (S.H.); weijiepeng@126.com (W.P.)

**Keywords:** capsaicin, type 1 diabetes mice, gut microbiome, anxiety, depression, cognitive decline

## Abstract

**Background/Objectives**: Diabetes mellitus is often accompanied by mental health complications, including anxiety, depression, and cognitive decline. Recent research suggested that capsaicin, the active component of chili peppers, may influence mental health. This study aimed to determine the effect of dietary capsaicin on mental disorders in a type 1 diabetes (T1D) mouse model, while also exploring the potential involvement of the microbiota-gut-brain axis. **Methods**: We induced T1D in mice using streptozotocin (STZ) and administered a diet supplemented with 0.005% capsaicin for five weeks. Behavioral assessments, including the open field test (OFT), tail suspension test (TST), forced swimming test (FST), elevated plus maze (EPM) test, and Morris water maze (MWM) test, were conducted to evaluate depressive and anxiety-like behaviors as well as cognitive function. Targeted and untargeted metabolomics analyses were performed to assess neurotransmitter levels in the hippocampus and serum metabolites, while 16S rRNA sequencing was utilized to analyze gut microbiota composition. Intestinal barriers were determined using western blot detection of the tight junction proteins ZO-1 and occludin. **Results**: Dietary capsaicin exacerbated anxiety and depressive-like behaviors along with cognitive declines in T1D mice. Capsaicin reduced gut microbiota diversity and levels of beneficial bacteria, while broad-spectrum antibiotic treatment further intensified anxiety and depression behaviors. Metabolomic analysis indicated that capsaicin disrupted metabolic pathways related to tryptophan and phenylalanine, leading to decreased neuroprotective metabolites, such as kynurenic acid, hippurate, and butyric acid. Additionally, capsaicin diminished the expression of ZO-1 and occludin, indicating increased intestinal permeability. **Conclusions**: Dietary capsaicin aggravates gut microbiota and metabolic disturbances in diabetic mice, thereby worsening anxiety, depression, and cognitive decline.

## 1. Introduction

The global prevalence of diabetes mellitus (DM) has reached alarming levels, affecting over 463 million individuals as of 2019, with projections suggesting that this number could rise to 700 million by 2045 [1]. Diabetes impacts more than just physical health. It is often linked to a high prevalence of psychological disorders, especially anxiety and depression [2]. Epidemiological studies reveal that 30% to 40% of diabetic patients experience moderate to severe levels of depression, anxiety, or both. The risk of developing these emotional disorders is approximately twice as high in diabetic patients compared to those without diabetes [3]. Diabetics with emotional disorders often exhibit poor adherence to treatment, which leads to poorer glycemic control, increased healthcare costs, and a diminished quality of life. Despite advancements in diabetes management, including pharmacological interventions and lifestyle modifications, the psychological aspects of diabetes remain inadequately addressed.

Recently, the role of the microbiota-gut-brain axis in maintaining mental health has gained considerable interest. Increasing evidence shows that gut microbiota dysbiosis is associated with anxiety and depression [4]. A recent case-control study reported that T1D patients with depression exhibited distinct profiles of gut microbiota and serum metabolites [5], suggesting a possible involvement of the gut microbiota in diabetes with depression.

Dietary patterns are among the main external factors influencing gut microbiome structure and function, indirectly modulating the host’s mental health. Spicy diets are prevalent in various cultures worldwide, with an estimated 20% of the global population regularly consuming chili peppers. Recently, the health implications of spicy food consumption have attracted increasing attention. Capsaicin, the primary active component in chili peppers, is responsible for their spicy flavor. Research has indicated that dietary capsaicin can lower plasma glucose levels while enhancing insulin levels and sensitivity [6]. This positive impact on glucose regulation in obese diabetic ob/ob mice is likely linked to changes in gut microbiota composition, which increases the production of short-chain fatty acids, regulates gastrointestinal hormones, and reduces pro-inflammatory cytokines [6]. Additionally, potential benefits of capsaicin in central nervous system disorders, such as Alzheimer’s disease [7] and Parkinson’s disease, have been recognized [8]. However, the effects of capsaicin intake on mood disorders, including depression and anxiety, is complex and sometimes contradictory. Several studies have reported antidepressant effects of capsaicin in animal models [8,9], such as in a lipopolysaccharide-induced depression mouse model in which dietary capsaicin (0.005% capsaicin added to laboratory chow) influenced gut microbiota structure and numbers, thereby helping to prevent depression [9]. However, a recent dietary survey involving 1771 Chinese university students found a positive correlation between spicy food consumption and symptoms of depression and anxiety [10]. This inconsistency underscores the necessity for further research to clarify the effects of capsaicin on emotional health.

Therefore, this study aimed to investigate how dietary capsaicin affects depression and anxiety-like behaviors in a T1D mouse model, while also exploring the potential role of the microbiota-gut-brain axis in these effects.

## 2. Materials and Methods

### 2.1. Preparation of Diabetic Mice

Six-week-old male C57BL/6J mice weighing 20 ± 2 g were purchased from GemPharmatch Co. Ltd. (Nanjing, China) and housed in the Animal Center of Nanchang University. The experimental protocols were reviewed and approved by the animal ethics committee of Nanchang University (NCULAE-20240717001).

The T1D mouse model was established by intraperitoneal injection of 50 mg/kg streptozotocin (STZ, dissolved in 0.1 M citrate buffer, pH~4.8) for 5 consecutive days. Only the mice with fasting blood glucose level higher than 11.1 mM for two consecutive days were used as the diabetic model for the trials.

### 2.2. Experimental Protocols

The mice were randomly assigned to three groups: (1) Control group (CON, n = 7), injected with citrate buffer and fed standard laboratory chow; (2) Diabetic mice group (DM, n = 8), consisting of STZ-induced diabetic mice on standard laboratory chow; (3) Diabetes mice plus dietary capsaicin group (DM + CAP, n = 8), diabetic mice fed standard laboratory chow with 0.005% capsaicin for 5 weeks. Body weight, feed intake, and fasting glucose were measured weekly throughout the study. After the behavioral experiments, fecal samples of all mice were collected sterilely and stored at −80 °C for 16S sequencing. Then the mice were sacrificed, and the hippocampus and serum of each mouse were collected for metabolomics analysis. Intestinal tissue was collected for western blot analysis.

**Figure 1:**
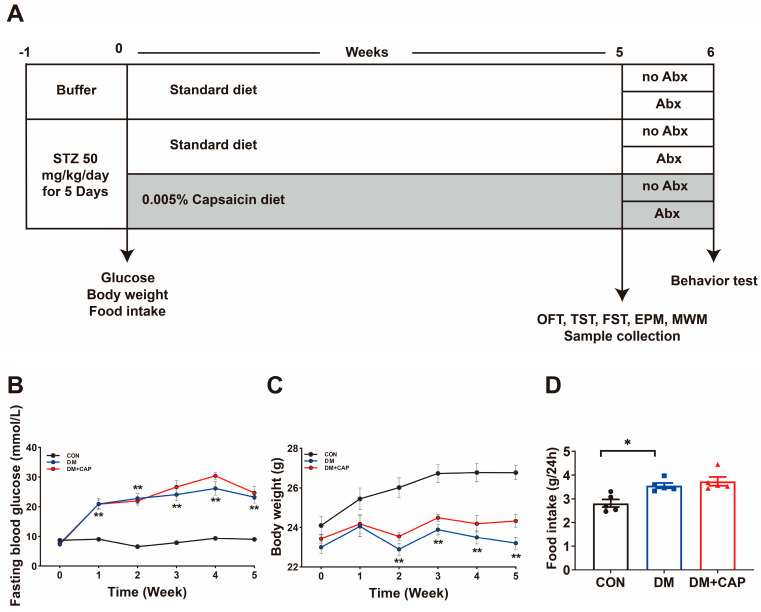
Effects of dietary capsaicin on the general condition of STZ-induced type 1 diabetes Mice (DM). (**A**) The schematic representation of the animal experiment. (**B**) Fasting blood glucose. (**C**) Body weight. (**D**) The average daily food intake. Data are presented as mean ± SEM (n = 7–8 mice); one-way ANOVA; post hoc Bonferroni test; * *p* < 0.05 and ** *p* < 0.01.

To evaluate the role of gut microbiota in anxiety and depressive-like behaviors, the mice were treated with a broad-spectrum antibiotic (Abx) cocktail (ampicillin; metronidazole; neomycin 1 mg/mL; vancomycin 0.5 mg/mL) in the drinking water for 1 week for intestinal flora depletion. The experimental procedure is shown in Figure 1B. After receiving either a standard diet or a capsaicin diet for 5 weeks, the mice of each group were sub-divided into Abx-treated and non-Abx-treated groups. OFT and FST were performed to compare the effects of Abx treatment and non-Abx treatment on anxiety/depressive-like behavior.

### 2.3. Behavior Tests

#### 2.3.1. Open Field Test (OFT)

The OFT was used to measure anxiety- and depressive-like behavior. To acclimate to the environment, animals were moved to the behavior room 1 h before initiating the test. The total moving distance of the mouse, the time spent in the center, the distance moved in the center, and the number of entries into the center were recorded within 5 min through the SMART3.0 digital tracking system (Panlab).

#### 2.3.2. Tail Suspension Test (TST)

The TST was utilized to assess behavior indicative of depression. After a 1 h acclimatization, a piece of adhesive tape was placed about 1 cm from the tip of the tail, with the mice’s head positioned 15 cm above the ground. The TST was performed for a 5 min and 15 s test period, and the immobility time was recorded for the last 5 min of the test.

#### 2.3.3. Forced Swimming Test (FST)

The FST was used to measure depressive-like behavior. After a 1 h acclimatization, the mice were placed in a transparent cylinder containing water (maintained at 22 °C and measuring 20 cm in depth). The FST was performed for a 5 min and 15 s test period, and the immobility time was recorded for the last 5 min of the test.

#### 2.3.4. Elevated Plus Maze Test (EPM)

Anxiety was measured using an EPM. After 1 h of acclimatization, mice were put into the center of the EPM, which had open arms and closed arms. The total distance traveled, time spent in the open arms, and distance traveled in open arms were recorded by the SMART3.0 digital tracking system (Panlab) in 5 min.

#### 2.3.5. Morris Water Maze Test (MWM)

The Morris water maze test (MWM) was performed to assess cognitive function. Briefly, each mouse was placed into a circular pool (diameter 150 cm, water temperature 22 °C) from four starting points and allowed to find the escape platform within 60 s. During this period, the escape time of each mouse was recorded. On the first day of the test (Day 0), the platform was placed 1 cm above the water surface. Subsequently, the platform was submerged below the water surface, and white dye was added to the water for four days of acquisition training (Days 1–4). Each mouse was allowed four trials per day, with at least 30 min between trials.

### 2.4. Targeted Metabolomics Identified the Neurotransmitters in Hippocampus

The hippocampal tissues of the mice were collected and sent to the Beijing Genomics Institute (BGI) in Shenzhen, China, for targeted LC/MS metabolomics. The tissue was weighed and then placed in a centrifuge tube with 50% methanol and magnetic beads for disruption. The mixture was centrifuged for 2 min at 25,000 rpm. Equal volumes of supernatant and standards, which were mixed with 39 neurotransmitters, were added to a centrifuge tube containing 50% methanol. A 20 μL aliquot of the supernatant was taken and added to the binding solution. The analysis was performed using a Waters I-class—AB Sciex 6500 liquid-mass tandem instrument (Waters, Milford, MA, USA), equipped with a Waters BEH C18 chromatographic column (1.7 μm × 2.1 × 100 mm, Waters, Milford, MA, USA). Data and differential analysis were performed using the R package metaX (version: 1.4.2).

### 2.5. The Gut Microbiota Composition of Mice Feces Determined by 16S rRNA Gene Sequencing

We collected about 50–100 mg of fecal samples from each mouse’s colon and promptly placed them in liquid nitrogen. The samples were then transported to the Beijing Genome Institute (BGI, Shenzhen, China) for 16S rRNA sequencing. Briefly, the V3V4 variable region of bacterial/fungal 16S rDNA was amplified by PCR reaction. DNA sequencing was performed using an Illumina PE300 (BGI, Shenzhen, China). Cutadapt v2.6 and Usearch (97% sequence similarity) were used to obtain clear data and OTUs. Sequences were matched against the RDP database (v2.2).

### 2.6. Serum Metabolomics Determined by Untargeted Metabolomics

Serum samples of the mice were sent to the Beijing Genomics Institute (BGI) (Shenzhen, China) for untargeted LC/MS metabolomics. Briefly, the melted serum samples were extracted with the extraction agent (methanol:acetonitrile:water = 4:2:1), centrifuged at 25,000× *g* for 15 min at 4 °C, and the supernatant was collected for detection. Metabolite separation and detection were performed using a Waters UPLC I-Class Plus (Waters, Milford, MA, USA) in tandem with a Q Exactive high-resolution mass spectrometer (Thermo Fisher Scientific, Waltham, MA, USA). Mass spectrometry data were collected using a Q Exactive mass spectrometer (Thermo Fisher Scientific, USA), and the data were imported into Compound Discoverer 3.3 (Thermo Fisher Scientific, USA) software, then combined with the BMDB (BGI Metabolome Database), mzCloud database, and ChemSpider online database for mass spectrometry data analysis. The expression data were analyzed using the limma package from R and false discovery rate (FDR) control for statistical assessment of the data (corrected *p* < 0.05 was considered significant).

### 2.7. Western Blot

Intestinal tissue samples were washed twice with PBS. Subsequently, 40 mg of tissue was weighed and homogenized in protein lysate. The protein concentration was determined using a Bicinchoninic Acid Assay (BCA) protein assay kit.

Equal amounts of protein were loaded into 10% SDS-PAGE gels and then transferred to PVDF membranes. The membranes were blocked for 2 h with 8% non-fat milk and incubated overnight at 4 °C with primary antibodies against ZO-1 (Proteintech Group, Wuhan, China), Occludin (Proteintech Group, Wuhan, China), and β-actin (Solarbio science & technology Co., Ltd., Beijing, China) at 4 °C overnight. The membranes were then incubated with appropriate dilutions of HRP-conjugated goat anti-rabbit or goat anti-mouse secondary antibodies at room temperature for 2 h. The membranes were then washed five times and a signal developed by enhanced chemiluminescence. Assays were conducted in triplicate.

### 2.8. Statistical Tests

Statistical analyses were performed using GraphPad Prism 9 and R version 4.4.0.

- Results are presented as mean ± SEM and analyzed via one-way ANOVA (post hoc Bonferroni test) and a Kruskal–Wallis test (Dunn’s post-hoc test).
- Data visualization was accomplished using the following software: GraphPad Prism 9, heatmap package, and the ggplot2 package of R language.
- For details on sample sizes, effect sizes, confidence intervals, and *p*-values, please refer to Supplementary Materials Table S1.

## 3. Results

### 3.1. Effects of Dietary Capsaicin on the General Condition of STZ-Induced Type 1 Diabetes Mice

We created STZ-induced type 1 diabetes mellitus (DM) mice and supplemented their diet with 0.005% capsaicin for 5 weeks (Figure 1A). Weekly measurements showed that DM mice displayed typical symptoms of diabetes: elevated fasting blood glucose levels, reduced body weight, and increased daily food intake (*p* < 0.05; Figure 1B–D). Dietary capsaicin did not have a significant effect on blood glucose levels, daily food intake, or body weight in DM mice (*p* > 0.05; Figure 1B–D).

### 3.2. Dietary Capsaicin Aggravates Anxiety and Depressive-like Behaviors and Cognitive Decline in Diabetic Mice

**Figure 2:**
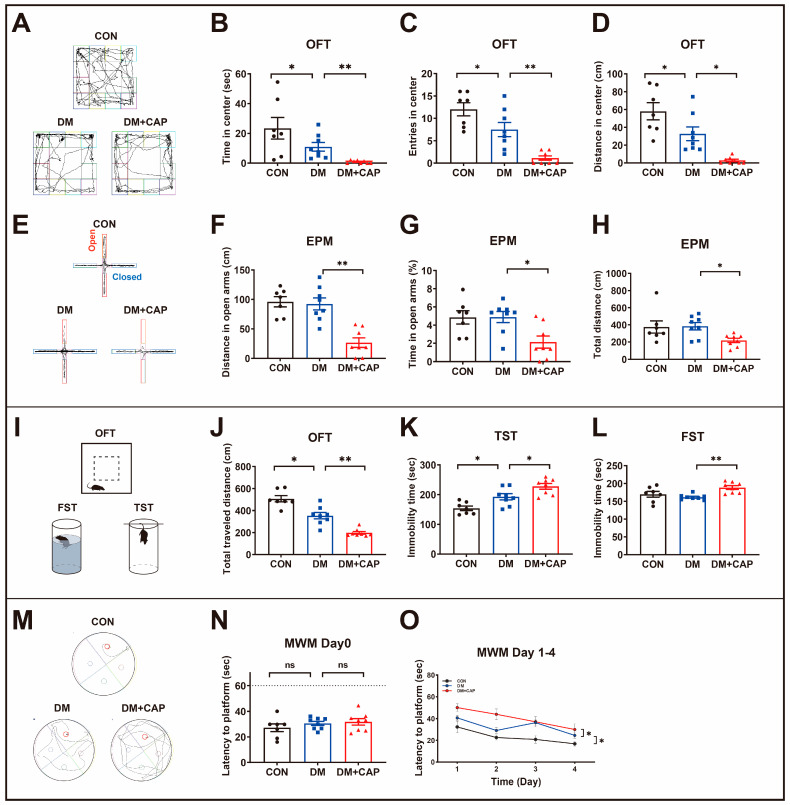
(**A**–**D**) The representative movement tracks, time spent in the center, traveled distance in the center and entries into the center in the open field test (OFT). (**E**–**H**) The representative movement tracks, total distance, distance traveled, and percentage of time spent on the open arms in elevated plus maze test (EPM). (**I**–**L**) The total traveled distance in the OFT, the immobility time in the forced swimming test (FST), and the immobility time in the tail suspension test (TST). (**M**–**O**) Cognitive Function: The representative movement tracks, learning phase, and 4 day memory retention phase of the Morris water maze test (MWM). All the values are expressed as mean ± SEM (n = 7–8 mice); one-way ANOVA; post hoc Bonferroni test; * *p* < 0.05 and ** *p* < 0.01.

We conducted several behavioral tests to investigate how dietary capsaicin affects the mental state and cognitive function of DM mice. Firstly, the open field test (OFT) and elevated plus maze (EPM) test were conducted to examine anxiety-like behaviors of mice. DM mice showed fewer exploratory behaviors in the OFT (Figure 2B–D) compared to CON mice. Specifically, they spent less time and traveled shorter distances in the center (*p* < 0.05), and made fewer entries into the center (*p* < 0.05). Dietary capsaicin further reduced the time spent (*p* < 0.01), distance traveled (*p* < 0.05, Figure 2D), and number of entries into the center (*p* < 0.01) in the OFT compared to DM mice. The EPM test (Figure 2F–H) further indicated that capsaicin significantly reduced both the distance traveled (*p* < 0.01) and the time spent (*p* < 0.05) in the open arms, as well as the total distance traveled (*p* < 0.05) when compared to the DM group. These results suggest that dietary capsaicin aggravates anxiety-like behaviors in DM mice.

Additionally, we assessed depressive-like behaviors using the OFT (Figure 2J), tail suspension test (TST, Figure 2K), and forced swimming test (FST, Figure 2L). Compared to CON mice, DM mice showed a decreased total traveled distance in the OFT (*p* < 0.05) and an increased immobility time in the TST (*p* < 0.05), suggesting that DM increased depression-like behaviors. Furthermore, DM + CAP mice exhibited more severe depressive behaviors than DM mice, as shown by a decreased total traveled distance (*p* < 0.01) in the OFT and increased immobility time in both the TST (*p* < 0.05) and FST (*p* < 0.01).

Cognitive function was assessed using the Morris water maze (MWM). On Day 0 of the learning phase, the three groups showed no significant differences (*p* > 0.05, Figure 2N). During the memory retention phase (days 1–4, Figure 2O), the DM mice took longer to escape and find the hidden platform (*p* < 0.05) compared to the CON mice, while dietary capsaicin further prolonged this latency (*p* < 0.05). These findings suggest that dietary capsaicin worsens the cognitive decline associated with diabetes mellitus.

### 3.3. Targeted Metabolomics Identified the Differences in Neurotransmitter Levels in the Hippocampus Among the Groups

**Figure 3:**
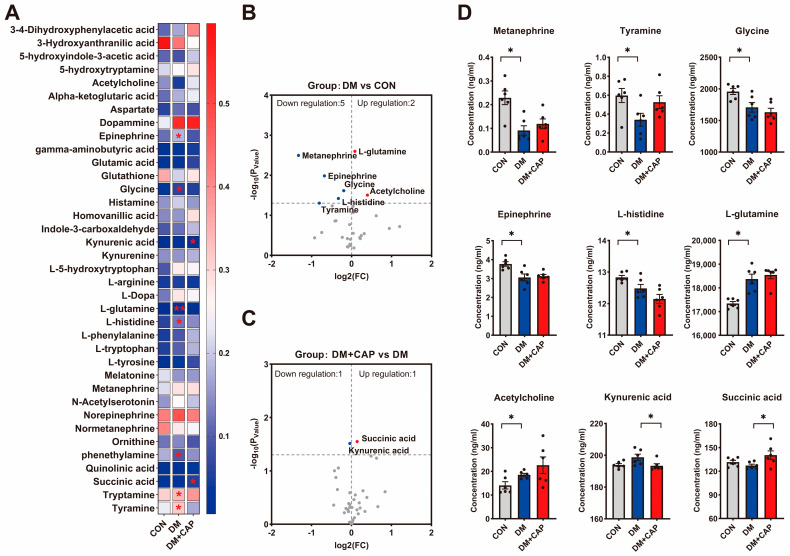
Targeted metabolomics identified the differential neurotransmitters in hippocampus among groups. (**A**) The heatmap of the differential neurotransmitters in the hippocampus. (**B**,**C**) Volcano plot of differential neurotransmitters in hippocampus, Red = up and blue = down. (**D**) Representative altered neurotransmitters. All the values are expressed as mean ± SEM (n = 6 mice); one-way ANOVA; post hoc Bonferroni test; * *p* < 0.05 and ** *p* < 0.01.

A targeted metabolomics analysis was performed to detect the levels of 37 neurotransmitters in the hippocampus of mice. The heatmap (Figure 3A) illustrates the differences in neurotransmitter content among the three groups. As shown in the volcano plot, nine out of the 37 neurotransmitters examined showed significant changes. Compared to the CON group, the DM group had five neurotransmitters upregulated and two downregulated. In contrast, the DM+CAP group had one neurotransmitter upregulated and one downregulated compared to the DM group (Figure 3B). Specifically, several neurotransmitters in the hippocampus of the DM mice, including epinephrine, metanephrine, tyramine, L-histidine, and glycine, were significantly reduced (*p* < 0.05, Figure 3D) compared to the CON mice. Conversely, the levels of L-glutamine and acetylcholine were elevated (*p* < 0.05, Figure 3D). Dietary capsaicin did not reverse these changes in neurotransmitter levels in the hippocampus of the DM mice. Instead, it led to a reduction in kynurenic acid and an increase in succinic acid levels (*p* < 0.05, Figure 3D).

### 3.4. Dietary Capsaicin Exacerbated the Gut Microbiota Imbalance in DM Mice

**Figure 4:**
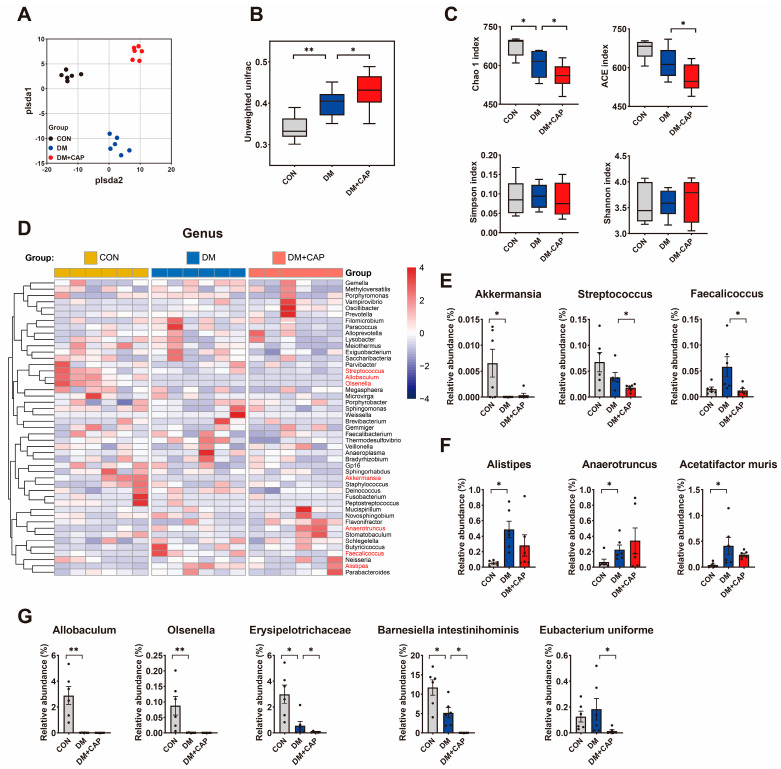
Effect of dietary capsaicin on the gut microbiota in DM mice based on 16S rRNA gene analysis. (**A**) Partial least squares discriminant analysis (PLSDA) for 16S sequencing data. (**B**) Box diagram based on unweighted UniFrac beta diversity. (**C**) The alpha-diversity indices including the Chao1 index, ACE index, Simpson index, and Shannon index. (**D**) Heatmap of gut microbiota composition at the genus level. (**E**) Relative abundance of beneficial bacteria (*Akkermansia*, *Streptococcus*, *Faecalicoccus*). (**F**) Relative abundance of harmful bacteria (*Alistipes*, *Anaerotruncus*, *Acetatifactor muris*). (**G**) Relative abundance of SCFA–producing bacteria (*Allobaculum*, *Olsenella*, *Erysipelotrichaceae*, *Barnesiella intestinihominis*, *Eubacterium uniforme*). All the values are expressed as mean ± SEM (n = 6 mice); Kruskal–Wallis test followed by Dunn’s post hoc test (* *p*-value < 0.05, ** *p*-value < 0.01).

We used 16S rRNA gene sequencing to analyze the composition, abundance, and function of gut microbiota in mouse fecal samples. The β-diversity indices, including partial least squares discriminant analysis (PLS-DA) (Figure 4A) and unweighted UniFrac, revealed distinct clustering of microbiota compositions, indicating significant differences in the gut microbiota community among the three groups. The α-diversity indices, Chao1 (*p* = 0.00951) and Ace (*p* = 0.01383), indicated that the species richness of gut microbiota decreased significantly in the DM group and further decreased in the DM+CAP group, while Shannon (*p* = 0.89484) and Simpson (*p* = 0.79607) indicated no significant difference in the diversity of gut bacteria among the three groups (Figure 4C).

Hierarchical clustering with a heatmap shows the relative abundance of representative changes at the genus level (Figure 4D). The relative abundance of well-known beneficial bacteria, such as *Akkermansia* (*p* < 0.05, Figure 4G) and several short-chain fatty acids (SCFAs)-producing bacteria, such as *Allobaculum*, *Olsenella*, *Erysipelotrichaceae*, and *Barnesiella intestinihominis* (*p* < 0.05, Figure 4G), was significantly reduced in DM mice, while certain harmful bacteria, including *Alistipes*, *Anaerotruncus*, and *Acetatifactor muris*, were markedly increased. Compared to the DM group, dietary capsaicin not only failed to reduce harmful bacteria levels but also further decreased the abundance of certain beneficial bacteria, like *Streptococcus* and *Faecalicoccus* (*p* < 0.05, Figure 4F). Additionally, the levels of SCFAs-producing bacteria, including *Erysipelotrichaceae*, *Barnesiella intestinihominis*, and *Eubacterium uniforme* (*p* < 0.05, Figure 4G), were also lowered.

### 3.5. Effects of Dietary Capsaicin on Serum Metabolomics in DM Mice

**Figure 5:**
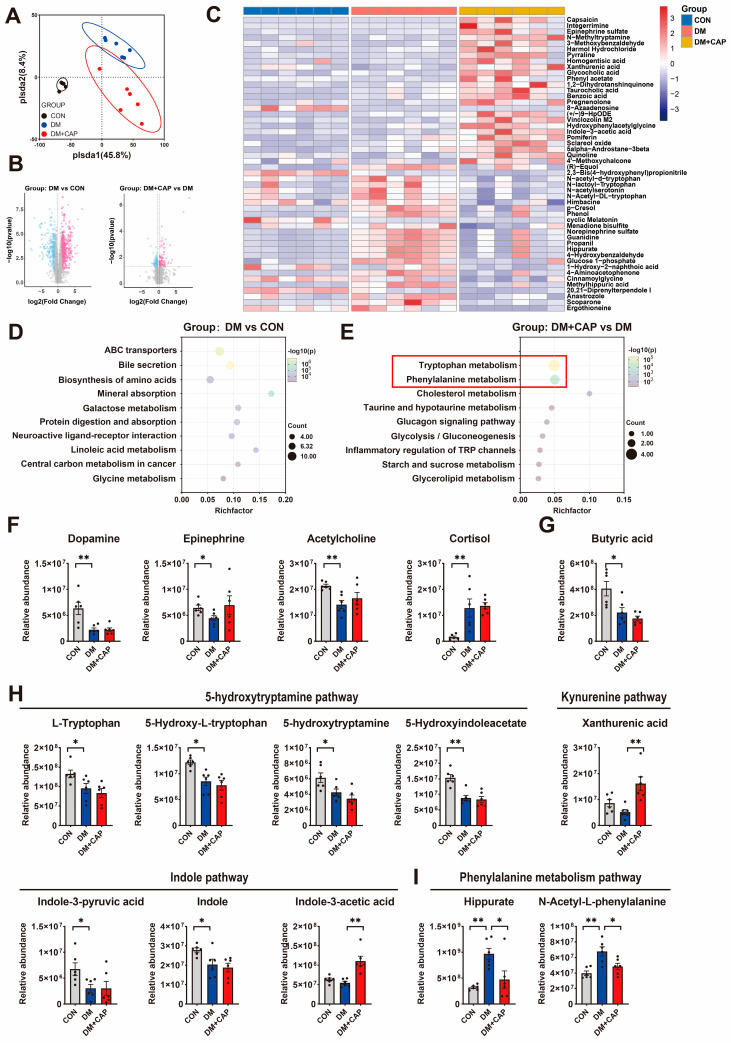
Serum metabolomics detected by untargeted metabolomics. (**A**) Partial least squares discriminant analysis (PLSDA) for metabolomic data. (**B**) Volcano plot of differentially expressed metabolites in serum, Red = up and blue = down. (**C**) Heatmap of the top 25 up- and 25 down- regulated (fold change) serum metabolites between different groups. (**D**,**E**) KEGG pathway analysis. (**F**) Representative transmitters and hormones with significant differences in serum. (**G**) Levels of butyric acid. (**H**) Representative metabolites in three pathways of tryptophan metabolism. (**I**) Representative metabolites in phenylalanine metabolism pathway. All the values are expressed as mean ± SEM (n = 6 mice); Kruskal–Wallis test followed by Dunn’s post hoc test (* *p*-value < 0.05, ** *p*-value < 0.01).

Furthermore, we used untargeted metabolomics to analyze changes in serum metabolites in diabetic mice and assess the effects of dietary capsaicin. Partial least squares discriminant analysis (PLS-DA) indicated that the CON, DM, and DM + CAP groups were distinct and had minimal overlap. This suggests that each group has a unique metabolomic profile (Figure 5A). The volcano plot analysis revealed 478 up- and 256 downregulated in the DM group compared to the CON group, and 83 up- and 70 downregulated in the DM+CAP group compared to the DM group (*p* < 0.05, |FC| > 1.2, Figure 5B). The clustering heatmap revealed the clustering analysis of differential metabolites among the three groups (Figure 5C).

Specifically, in the DM group, the relative abundances of neurotransmitters such as dopamine, serotonin (5-HT), adrenaline, and acetylcholine (Ach) were significantly lower compared to the CON group. Meanwhile, the level of cortisol, a stress hormone, was significantly higher (Figure 4D). Dietary capsaicin not only did not improve the alterations in neurotransmitter levels, it also appeared to further decrease 5-HT levels. Furthermore, the DM group exhibited a significant decrease in butyrate, a key SCFA linked to gut health (*p* < 0.05, Figure 5E). Capsaicin also appeared to further reduce the levels of butyrate, although these differences did not reach significance.

The Kyoto Encyclopedia of Genes and Genomes (KEGG) pathway analysis demonstrated that in the “DM vs. CON” group, the differential metabolites were significantly enriched in various metabolic pathways, including ABC transport proteins, bile secretion, amino acid biosynthesis, mineral absorption, and galactose metabolism (Figure 5D). In the “DM + CAP vs. DM” group, the metabolic pathways of tryptophan and phenylalanine displayed the highest levels of significance.

Compared to the CON group, the DM mice exhibited significantly reduced levels of metabolites in both the tryptophan-5-Hydroxytryptamine pathway, including L-Tryptophan, 5-Hydroxy-L-tryptophan (5-HTP), 5-Hydroxytryptamine (5-HT) and 5-HydroxyIndoleacetate (5-HIAA), and the tryptophan-indolic pathway, including Indole-3-pyruvic (IPA) and Indole (*p* < 0.05, Figure 5G,H). Dietary capsaicin increased the level of Indole-3-acetic acid (IAA) in the tryptophan-indolic pathway and Xanthurenic (XA) of the tryptophan-kynurenine pathway as compared with DM mice. Hippurate, a metabolite from phenylalanine metabolism, showed a negative correlation with depression. In this study, the DM mice had higher levels of hippurate compared to the CON mice (*p* < 0.01, Figure 5I). However, dietary capsaicin significantly reduced hippurate levels in the DM mice.

### 3.6. Correlations Between Gut Microbes, Serum Metabolites and Hippocampal Neurotransmitters

**Figure 6:**
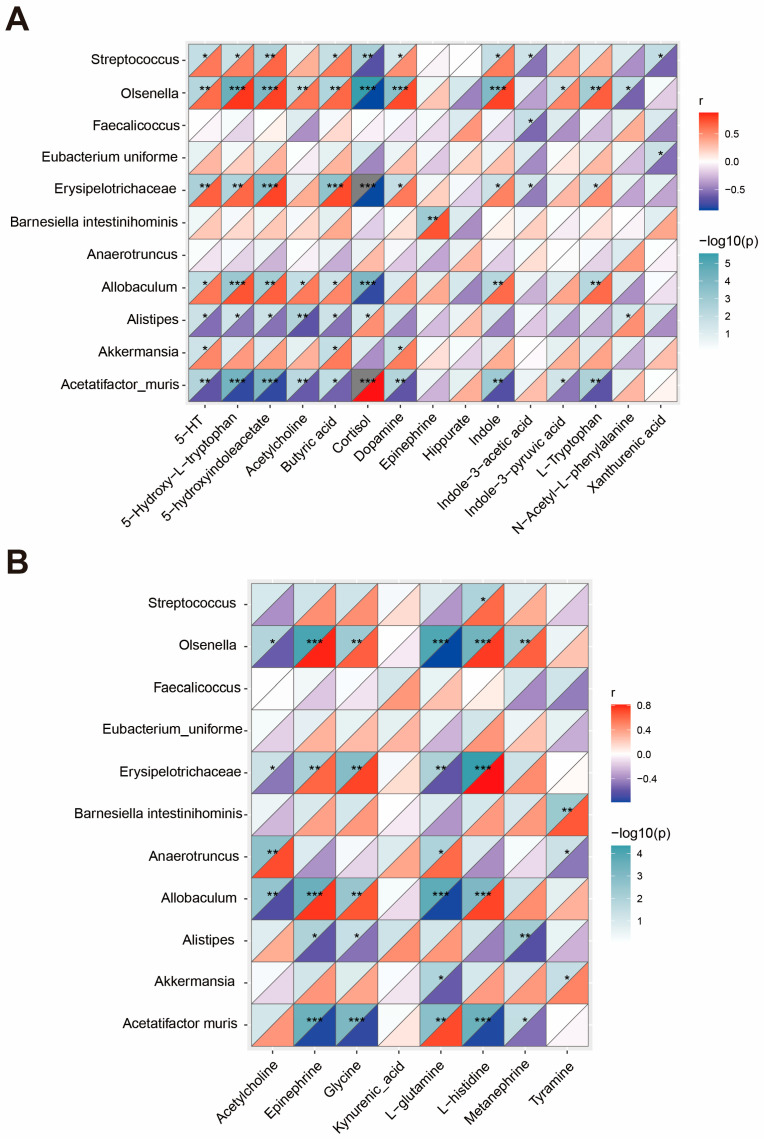
Spearman correlation analysis of gut microbiota, serum metabolites, and hippocampal neurotransmitters. (**A**) The Spearman’s rank correlation coefficient between gut differential microbiota and serum differential metabolites. (**B**) The Spearman’s rank correlation coefficient between gut differential microbiota and hippocampal differential neurotransmitters. * *p* < 0.05, ** *p* < 0.01, and *** *p* < 0.001.

We performed a Spearman’s correlation analysis to determine how different gut bacteria are associated with changes in serum metabolites and hippocampal neurotransmitters (Figure 6A,B).

*Allobaculum*, *Olsenella*, *Erysipelotrichaceae*, and *Akkermansia* were significantly reduced in the DM group compared to the CON group. These microbiota showed positive correlations with serum levels of 5–HT, dopamine, and butyric acid, while exhibiting negative correlations with cortisol in serum. *Allobaculum*, *Olsenella*, and *Erysipelotrichaceae* also displayed positive correlations with multiple neurotransmitters in the hippocampus, including epinephrine, glycine, L–histidine, and negative correlations with Ach and L–glutamine in the hippocampus. *Akkermansia* exhibited positive correlations with tyramine and negative correlations with L–glutamine in the hippocampus. In contrast, *Alistipes* and *Acetatifactor muris*, which were markedly increased in the DM mice, showed positive correlations with cortisol in serum and negative correlations with serum levels of 5–HT, dopamine, and butyric acid. They also exhibited negative correlations with levels of epinephrine, glycine, L–histidine, and metanephrine in the hippocampus.

As previously noted, dietary capsaicin notably reduced the relative abundance of several beneficial bacteria in the DM mice, such as *Streptococcus*, *Erysipelotrichaceae*, and *Barnesiella intestinihominis*. *Streptococcus* and *Erysipelotrichaceae* showed positive correlations with tryptophan metabolites (5–HT, 5–HTP, 5–HIAA, Indole, IAA), dopamine, and butyric acid, but negative correlations with cortisol, IAA, and XA. *Barnesiella intestinihominis* exhibited positive correlations with serum epinephrine and hippocampal tyramine.

### 3.7. Effect of Dietary Capsaicin on the Intestinal Permeability in DM Mice

**Figure 7:**
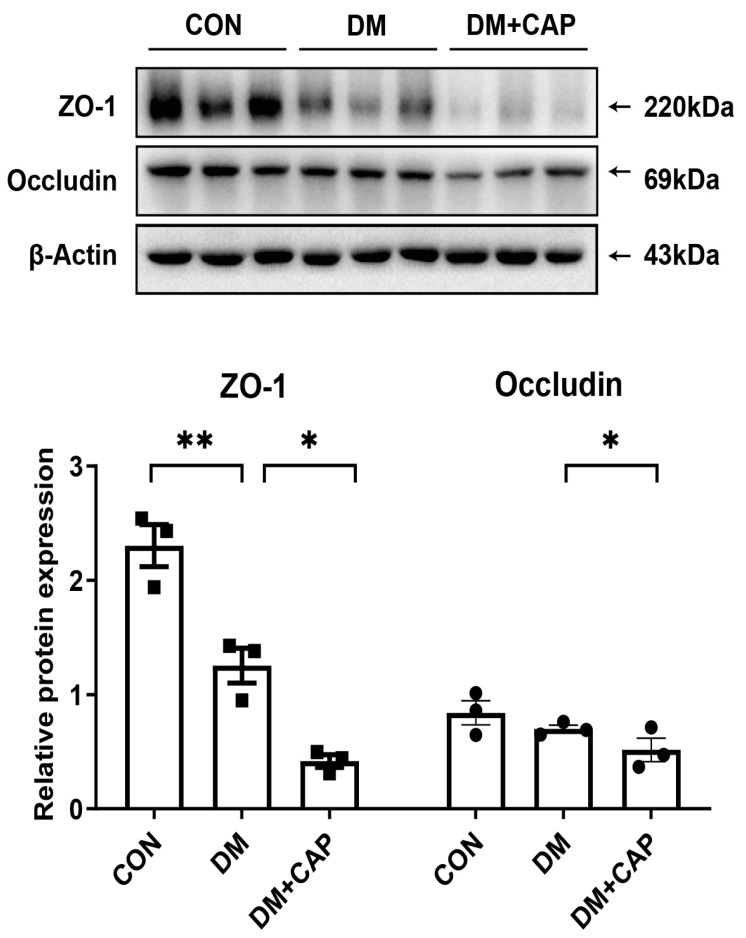
Effect of dietary capsaicin on the expression of intestinal tight junction protein ZO-1 and occludin in DM mice. Effect of dietary capsaicin on the expression of intestinal tight junction protein ZO-1 and occludin in DM mice. All the values are expressed as mean ± SEM (n = 3 mice); one-way ANOVA; post hoc Bonferroni test; * *p* < 0.05 and ** *p* < 0.01.

We investigated the effect of dietary capsaicin on intestinal permeability by measuring the protein levels of the tight junction proteins ZO-1 and occludin in intestinal tissues. Western blotting results revealed that the expression of ZO-1 was significantly lower in the DM mice compared to the CON mice (*p* < 0.01, Figure 7). Furthermore, the DM + CAP group displayed even lower levels of ZO-1 and occludin, indicating that intestinal permeability was increased in the DM mice, and dietary capsaicin further impaired intestinal barrier function.

### 3.8. Gut Microbiota Depletion Exacerbates Anxiety and Depressive-like Behaviors in DM Mice with Capsaicin Diet

**Figure 8:**
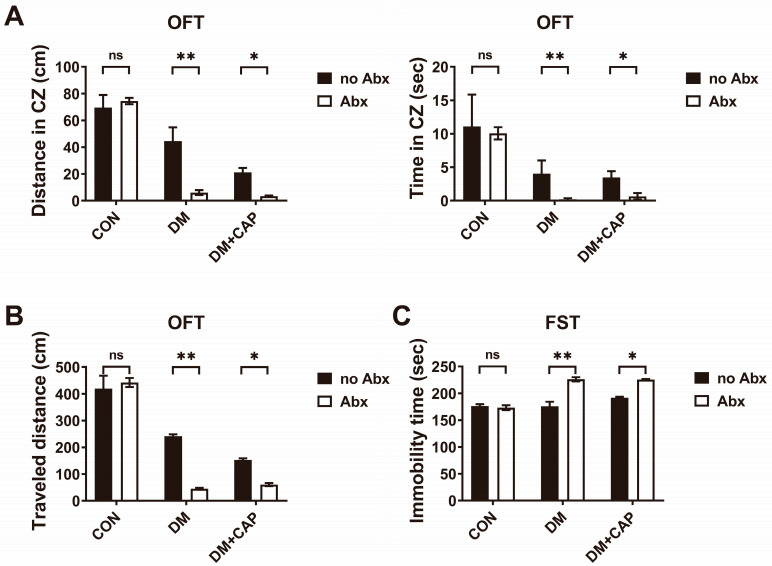
Gut microbiota depletion exacerbates anxiety and depressive-like behaviors in DM mice with capsaicin diet. (**A**) The time spent in the center and traveled distance in the center in the open field test (OFT). (**B**) The total traveled distance in OFT. (**C**) The immobility time in the forced swimming test (FST). All the values are expressed as mean ± SEM (n = 3 mice); one-way ANOVA; post hoc Bonferroni test; * *p* < 0.05 and ** *p* < 0.01.

Additionally, we administered Abx to determine whether gut microbiota depletion impacts anxiety and depressive-like behaviors in DM mice. Figure 1B illustrates the experimental procedure, indicating that half of the mice in each group received Abx in their drinking water for one week. Compared to their no-Abx controls, Abx treatment increased anxiety behaviors in both the DM mice and the DM+CAP mice, as evidenced by less time spent and shorter distances traveled in the center zone (*p* < 0.01, *p* < 0.05, respectively), as well as fewer entries into the center (*p* < 0.01) in OFT (Figure 8A). Moreover, depressive-like behaviors worsened following Abx treatment in both the DM mice and the DM + CAP mice. This was indicated by a decreased total distance traveled in the OFT (*p* < 0.01, Figure 8B) and increased immobility time in the FST (*p* < 0.01, Figure 8C). However, Abx did not significantly affect anxiety or depressive behaviors in normal mice.

## 4. Discussion

Diabetes mellitus is frequently accompanied by mental complications, particularly depression and anxiety, which significantly impact the patient’s quality of life and disease management. Many dietary components, such as fruits, vegetables, dairy products, and medicinal herbs, have been shown to help protect against mental disorders by regulating the gut microbiota [11]. Previous studies indicate that administering capsaicin either intraperitoneally or intracerebroventricularly has an antidepressant effect in some animal models. In contrast to these injection methods, dietary supplementation with capsaicin is more similar to how humans consume spicy foods. Recent study has shown that a four-month diet of 0.005% capsaicin in laboratory chow can prevent depression-like behaviors induced by lipopolysaccharide (LPS). To investigate the potential benefits of spicy food consumption on mental disorders in individuals with diabetes, we observed the effects of a 0.005% capsaicin diet in a mouse model of type 1 diabetes (T1D). This dosage is comparable to the level of a high capsaicin diet among humans.

In the present study, mice treated with STZ exhibited typical symptoms of diabetes, including elevated blood glucose levels, increased daily food intake, and decreased body weight. Dietary capsaicin (0.005%) administered for 5 weeks did not significantly affect fasting blood glucose, food intake, or body weight. Although research on other diabetic animal models suggests that capsaicin has positive effects on glucose metabolism [12], human studies show conflicting results, indicating that capsaicin does not affect blood glucose or insulin levels [13]. The dose and method of capsaicin ingestion could contribute to the heterogeneity observed between studies.

As shown in the behavioral experiment, T1D mice exhibited pronounced anxiety and depressive-like behaviors, characterized by reduced exploration and distance traveled in the OFT and EPM tests, as well as increased immobility in the TST. Additionally, the MWM results reveal prolonged escape latency to find the hidden platform, reflecting cognitive deficits. These observations align with previous studies that animal models of diabetes often exhibit a phenotype characterized by depression-like and/or anxiety-like behaviors and cognitive decline.

While previous animal studies indicating that dietary capsaicin alleviated LPS-induced depressive behaviors and mitigated cognitive decline in mouse models of Alzheimer’s disease, the human studies provide conflicting results. Notably, a 15-year Open Cohort Study indicated a positive correlation between higher chili intake and cognitive decline among Chinese adults, suggesting potential adverse effects of capsaicin consumption in humans [14]. Furthermore, a recent cross-sectional survey among university students suggested a positive association between frequent spicy food consumption and depression and anxiety symptoms [10]. Our findings are consistent with these human studies, showing that dietary capsaicin worsens anxiety, depressive-like behaviors, and cognitive decline in diabetic mice. This was evidenced by significant reductions in locomotor and exploratory activities, increased immobility, and prolonged escape latencies. Such contradictory outcomes suggest that the impact of spicy diets or capsaicin on mental health may vary across different disease models. The differences in timing and duration of capsaicin exposure may also elucidate the contrasting outcomes observed.

Anxiety and depression are linked to neurotransmitter dysfunction in the hippocampus, a crucial area for regulating stress and emotional responses. We employed targeted metabolomics to detect the levels of 37 neurotransmitters within the hippocampus of mice. Compared to the CON mice, the levels of epinephrine, metanephrine, tyramine, L-histidine, and glycine in the DM mice were significantly reduced, while the levels of Ach and L-glutamine increased. According to the cholinergic-adrenergic theory, depression arises when cholinergic signaling surpasses noradrenergic tone [15]. Ach signaling in the hippocampus promotes behaviors related to anxiety and depression [16]. Therefore, the rise in Ach levels and the drop in epinephrine may contribute to anxiety and depression-like behaviors in the DM mice. Interestingly, dietary capsaicin did not reverse the alteration of neurotransmitter levels in the hippocampus of the DM mice. Instead, it led to a further increase in Ach levels and a reduction in kynurenic acid. Kynurenic acid is an important metabolite in the kynurenine pathway of tryptophan metabolism. In the brain, kynurenic acid acts as a neuronal N-methyl-D-aspartate (NMDA) receptor antagonist. At physiological concentrations, it is generally considered a neuroprotective factor [17]. Due to its anti-inflammatory and neuroprotective properties, kynurenic acid is thought to play a protective role in depression [18]. Decreased kynurenic acid in epileptic spasms has been identified as a biomarker indicating responsiveness to corticosteroids [19]. Thus, the reduction of kynurenic acid may partially explain why capsaicin aggravates the anxiety and depressive-like behaviors in DM mice.

Recently, a growing number of studies have highlighted a potential mechanism linking microbiome dysbiosis to mental disorders and diabetes mellitus. A case-control study found that T1D patients with depression have distinct profiles of gut microbiota and serum metabolites [5]. Specifically, the bacteria *Alistipes*, *Phascolarctobacterium*, and *Butyricimonas* may predict the risk of T1D in individuals suffering from depression [5]. These findings suggest a possible involvement of gut microbiota in depression in people with diabetes. Using 16S rRNA sequencing, we found an imbalance of gut microbiota in STZ-induced DM mice, with the decrease of the species richness and beneficial bacteria (*Akkermansia*, *Allobaculum*, *Olsenella*, *Erysipelotrichaceae*, and *Barnesiella*), as well as an increase in harmful bacteria (*Alistipes*, *Anaerotruncus*, and *Acetatifactor muris*). *Akkermansia* is a well-known beneficial bacterium that alleviates depression-like behaviors and restored levels of 5-HT and dopamine [20]. *Alistipes* has been shown to be related to inflammation and mental health, which can hydrolyze tryptophan into indole, subsequently reducing 5-HT levels and exacerbating depression [21]. *Acetatifator muris* is considered a detrimental bacterium, associated with intestinal inflammation [22]. These findings provide further evidence that microbiome dysbiosis is involved in T1D.

The complex interplay between dietary capsaicin, gut microbiota, and disease progression has garnered considerable attention in recent years. Numerous studies suggest that capsaicin may help restore the balance of gut microbiota and provide health benefits, such as improving glucose homeostasis [6] and reducing obesity [23] and atherosclerosis [24]. However, emerging evidence also indicates that capsaicin may drive gut microbiota dysbiosis, potentially promoting cancer metastasis [25,26]. In this study, we found that dietary capsaicin decreased the species richness of gut microbiota in DM mice and reduced the levels of certain beneficial bacteria, including *Streptococcus*, *Faecalicoccus*, *Erysipelotrichaceae*, *Barnesiella intestinihominis*, and *Eubacterium uniforme*. *Streptococcus* is a beneficial bacterium with anti-inflammatory properties that can reverse depression-like behaviors caused by stress-altered gut microbiota [27]. *Erysipelotrichaceae*, *Barnesiella intestinihominis*, and *Eubacterium uniforme* are known to be SCFAs-producing bacteria [28,29,30]. These results suggest that dietary capsaicin exacerbates the gut microbiota disorder in DM mice.

The microbiota-gut-brain axis plays a crucial role in the communication between the gut and the brain, utilizing various pathways including microbial, nervous, endocrine, metabolic, and immune signals [4]. We employed untargeted metabolomics to detect the alteration in serum metabolites. The reduction of monoamine neurotransmitters such as 5-HT, norepinephrine, and dopamine has been identified as a primary contributor to depressive disorders. We found that the level of dopamine, 5-HT, adrenaline, and Ach in serum were significantly decreased in the DM group, alongside an elevation in cortisol, a hormone associated with stress response. Dietary capsaicin failed to ameliorate these alterations, and it may have exacerbated the decline in serotonin levels.

SCFAs are important metabolites produced by gut bacteria that have beneficial effects on brain function and mood. A decrease in SCFAs may result in impaired remyelination, increased inflammation, greater permeability of the blood-brain barrier and gut barrier, oxidative stress, and worsened depressive symptoms [31]. Butyrate, a major component of SCFAs, has been shown to exert antidepressant effects [32] and enhance the central expression of neurotrophic factors [31]. As mentioned above, the abundance of several SCFAs-producing bacteria was reduced in the DM and DM+CAP groups. Consistent with these results, butyric acid levels were significantly lower in the DM group and further decreased following capsaicin treatment.

KEGG pathway analysis revealed that the differential metabolites in the “DM + CAP vs. DM” group were significantly enriched in tryptophan metabolism and phenylalanine metabolism. The dysregulation of tryptophan metabolism has been associated to the pathophysiology of depression, anxiety, and cognitive impairments [33]. Tryptophan metabolism mainly involves three pathways: the 5-HT, the indolic, and the kynurenine pathway [34]. While the 5-HT pathway’s role in depression is well-documented, the involvement of metabolites in the other two tryptophan pathways is not fully elucidated. Our present study found that the level of metabolites of three tryptophan pathways in the DM mice were decreased when compared with the CON group. Dietary capsaicin cannot reverse these alterations, but it increased the levels of IAA in the indolic pathway and XA in the kynurenine pathway as compared with the DM mice. Phenylalanine is the precursor of the synthesis of dopamine, noradrenaline, and other neurotransmitters, which regulate emotions and stress. Thus, a normal phenylalanine metabolism is vital for mental health. Hippuric acid is a metabolite resulting from the microbial metabolism of phenylalanine [35]. Hippurate has been shown to be a general marker of metabolic health [36]. A recent Mendelian randomization analysis suggests that low hippurate levels may be the cause of an increased risk for depression [37]. In the present study, dietary capsaicin significantly decreased hippurate levels in the DM mice. Taken together, the results of serum metabolomics suggest that diabetes leads to abnormal serum metabolome, characterized by decreased levels of monoamine neurotransmitters and SCFA, as well as increased cortisol. These metabolic disturbances may underlie the comorbidity of anxiety and depression in DM. Dietary capsaicin not only fails to correct these metabolic disturbances but also exacerbates serum metabolism disorders by interfering with tryptophan and phenylalanine metabolism pathways.

Additionally, we applied a Spearman’s correlation analysis to explore the correlation between gut differential microbiota and serum differential metabolites. Specifically, the genera *Allobaculum*, *Olsenella*, *Erysipelotrichaceae*, *Akkermansia*, and *Streptococcus*, which were decreased in the DM and DM + CAP groups, correlate positively with serum levels of tryptophan metabolites, dopamine, and butyric acid, while exhibiting negative correlations with cortisol levels. Conversely, the increased abundance of *Alistipes* and *Acetatifactor muris* in diabetic mice correlates positively with elevated cortisol levels and negatively with serum levels of 5-HT, dopamine, and butyric acid. These findings suggest that gut microbiota dysbiosis may be a critical factor in metabolic disturbances in diabetic mice and dietary capsaicin.

The intestinal barrier function has been shown to play critical role in maintaining gut homeostasis. The dysregulation of tight junction proteins and increased intestinal permeability may facilitate the translocation of pro-inflammatory cytokines and microbial metabolites into the systemic circulation [38], thereby influencing neuroinflammatory pathways associated with depressive symptoms. ZO-1 and Claudins are the major types of intestinal epithelial tight junction proteins which form the intestinal barrier [39]. We observed that the expression of ZO-1 and occludin was significantly decreased in the DM mice, and capsaicin further decreased these two tight junction proteins, suggesting dietary capsaicin aggravated the destruction of intestinal barrier function in DM mice.

Furthermore, the present findings demonstrate that antibiotic (Abx) treatment, which effectively depleted gut microbiota, did not alter behaviors in normal mice but significantly intensified anxiety and depressive-like behaviors in both the DM and DM + CAP mice. This suggests that gut microbiota play a pivotal role in modulating mental health, particularly under metabolic stress. The observed exacerbation of behavioral symptoms post-Abx treatment may be linked to reduced levels of short-chain fatty acids and neurotransmitter precursors, which are crucial for mental well-being and are typically synthesized by gut microbiota.

Existing research has predominantly focused on the beneficial effects of dietary capsaicin on gut microbiota and health. Our study is the first to show that dietary capsaicin can aggravate mental disorders in type 1 diabetic mice through the microbiota-gut-brain axis, suggesting the dual-edged effects of capsaicin. However, this research has limitations due to its focus on only one dosage and the effects of capsaicin over 5–6 weeks. Future studies should explore varying dosages and the long-term impacts of capsaicin to gain a more comprehensive understanding of its potential in managing mood disorders associated with diabetes.

## 5. Conclusions

In conclusion, the current study demonstrates that dietary capsaicin fails to improve and instead exacerbates anxiety and depressive-like behaviors in a T1D mouse model. This detrimental effect of capsaicin is likely related to a further reduction of beneficial bacteria, which in turn interferes with the tryptophan and phenylalanine metabolism pathways, resulting in decreased levels of neuroprotective metabolites, such as kynurenic acid, hippurate, and butyric acid. This research suggests the potential risk of spicy diets amplifying anxiety and depression in diabetes patients, thereby providing new experimental evidence for dietary guidelines in managing diabetes-related mental health issues.

## Data Availability

Data from the study are available upon reasonable request.

## Supplementary Materials

The following supporting information can be downloaded at: https://www.mdpi.com/article/10.3390/nu17030593/s1, Table S1. Effects of dietary capsaicin on the general condition of STZ-induced type 1 diabetes mice. Table S2. Effects of dietary capsaicin on anxiety and depressive-like behaviors and Cognitive Function in DM mice. Table S3. Targeted metabolomics identified the differences in neurotransmitter levels in the hippocampus among the groups. Table S4. Dietary capsaicin exacerbated the gut microbiota imbalance in DM mice. Table S5. Serum metabolomics detected by untargeted metabolomics. Table S6. Effect of dietary capsaicin on the expression of intestinal tight junction protein ZO-1 and Occludin in DM mice. Table S7. Gut microbiota depletion exacerbates anxiety and depressive-like behaviors in DM mice with capsaicin diet.

## Abbreviations

The following abbreviations are used in this manuscript:

CAP	Capsaicin
DM	Diabetes mellitus
T1D	Type 1 diabetes
STZ	Streptozotocin
Abx	Antibiotic
OFT	Open field test
TST	Tail suspension test
FST	Forced swimming test
EPM	Elevated plus maze test
MWM	Morris water maze test
SCFAs	Short-chain fatty acids
5-HTP	5-Hydroxy-L-tryptophan
5-HIAA	5-hydroxyindoleacetate
XA	Xanthurenic acid
5-HT	Serotonin
IPA	Indole propionic acid
IAA	Indole-3-acetic acid
