# Supplementary material for: Dietary Capsaicin Exacerbates Gut Microbiota Dysbiosis and Mental Disorders in Type 1 Diabetes Mice
Source: Nutrients. 2025 Feb 6;17(3):593. doi: 10.3390/nu17030593 (PMC11821225; doi:10.3390/nu17030593)
Supplement: Supplementary file 1 [file nutrients-17-00593-s001.zip › nutrients-3430252-supplementary.pdf]

Statistical chart indicators include the group, sample size, mean, standard error of mean(SEM), standard deviation(SD), and confidence interval(CI).

Table S1. Effects of dietary capsaicin on the general condition of STZ-induced type 1 diabetes mice.

Indicators		Group CON (n=7)	DM (n=8)	DM+CAP (n=8)
Food intake	Mean	2.810	3.554	3.733
	SD	0.3646	0.2503	0.4106
	SEM	0.1631	0.1119	0.1836
	Lower 95% CI	2.357	3.243	3.223
	Upper 95% CI	3.263	3.865	4.243

Table S2. Effects of dietary capsaicin on anxiety and depressive-like behaviors and Cognitive Function in DM mice.

Indicators		Group CON (n=7)	DM (n=8)	DM+CAP (n=8)
Time in center in OFT	Mean	23.41	10.99	0.8550
	SD	19.16	8.131	0.9570
	SEM	7.243	2.875	0.3384
	Lower 95% CI	5.689	4.194	0.05489
	Upper 95% CI	41.13	17.79	1.655
Entries in center in OFT	Mean	12.00	7.500	1.125
	SD	3.873	4.504	1.356
	SEM	1.464	1.592	0.4795
	Lower 95% CI	8.418	3.735	-0.008814
	Upper 95% CI	15.58	11.27	2.259
Distance in center in OFT	Mean	58.00	32.66	2.831
	SD	25.46	21.65	3.732
	SEM	9.624	7.655	1.319
	Lower 95% CI	34.45	14.56	-0.2887
	Upper 95% CI	81.55	50.76	5.951
Distance in	Mean	95.93	92.24	26.40

open arms in EPM	SD	22.93	29.08	22.68
	SEM	8.668	10.28	8.020
	Lower 95% CI	74.72	67.93	7.438
	Upper 95% CI	117.1	116.5	45.37
Time in open arms in EPM	Mean	4.842	4.884	2.136
	SD	1.941	1.728	1.881
	SEM	0.7338	0.6108	0.6650
	Lower 95% CI	3.046	3.439	0.5639
	Upper 95% CI	6.637	6.328	3.709
Total disatance in EPM	Mean	374.6	383.1	218.2
	SD	187.7	123.0	71.23
	SEM	70.95	43.48	25.18
	Lower 95% CI	201.0	280.3	158.7
	Upper 95% CI	548.2	485.9	277.8
Total distance in OFT	Mean	507.3	353.3	197.2
	SD	76.13	82.12	34.22
	SEM	28.77	29.03	12.10
	Lower 95% CI	436.9	284.7	168.5
	Upper 95% CI	577.7	422.0	225.8
Immobility time in TST	Mean	154.2	192.6	227.8
	SD	20.25	29.46	26.71
	SEM	7.655	10.42	9.443
	Lower 95% CI	135.4	168.0	205.5
	Upper 95% CI	172.9	217.2	250.2
Immobility time in FST	Mean	169.7	161.6	188.6
	SD	21.25	7.418	15.15
	SEM	8.034	2.623	5.357
	Lower 95% CI	150.1	155.4	175.9
	Upper 95% CI	189.4	167.8	201.3
MWM Day0 Latency to platform	Mean	27.18	30.53	31.80
	SD	7.988	4.537	7.217
	SEM	3.019	1.604	2.552
	Lower 95% CI	19.79	26.74	25.77
	Upper 95% CI	34.57	34.33	37.83

Table S3. Targeted metabolomics identified the differences in neurotransmitter levels in the hippocampus among the groups.

Indicators		Group CON (n=6)	Group DM (n=6)	Group DM+CAP (n=6)
Metanephrine	Mean	0.2288	0.09118	0.1190
	SD	0.07052	0.04874	0.05078
	SEM	0.02879	0.01990	0.02073
	Lower 95% CI	0.1547	0.04003	0.06574
	Upper 95% CI	0.3028	0.1423	0.1723
Tyramine	Mean	0.5966	0.3411	0.5265
	SD	0.1823	0.1644	0.1658
	SEM	0.07442	0.06712	0.06769
	Lower 95% CI	0.4053	0.1685	0.3525
	Upper 95% CI	0.7879	0.5136	0.7005
Glycine	Mean	1957	1708	1630
	SD	113.3	185.9	152.2
	SEM	46.26	75.88	62.13
	Lower 95% CI	1839	1513	1471
	Upper 95% CI	2076	1903	1790
Epinephrine	Mean	3.770	3.058	3.126
	SD	0.2884	0.4482	0.2246
	SEM	0.1177	0.1830	0.09170
	Lower 95% CI	3.468	2.588	2.890
	Upper 95% CI	4.073	3.528	3.362
L-histidine	Mean	12.83	12.48	12.15
	SD	0.1505	0.3052	0.3489
	SEM	0.06145	0.1246	0.1424
	Lower 95% CI	12.68	12.16	11.78
	Upper 95% CI	12.99	12.80	12.51
L-glutamine	Mean	17342	18373	18546
	SD	199.8	508.5	387.6
	SEM	81.58	207.6	158.2
	Lower 95% CI	17133	17839	18139
	Upper 95% CI	17552	18906	18952

Acetylcholine	Mean	14.09	18.54	22.62
	SD	3.743	1.505	8.551
	SEM	1.528	0.6144	3.491
	Lower 95% CI	10.16	16.96	13.64
	Upper 95% CI	18.01	20.12	31.59
Kynurenic acid	Mean	193.9	198.7	193.4
	SD	2.116	4.793	3.160
	SEM	0.8640	1.957	1.290
	Lower 95% CI	191.7	193.7	190.1
	Upper 95% CI	196.1	203.7	196.7
Succinic acid	Mean	131.5	127.2	140.4
	SD	5.642	4.382	13.24
	SEM	2.303	1.789	5.407
	Lower 95% CI	125.6	122.6	126.5
	Upper 95% CI	137.5	131.8	154.3

Table S4. Dietary capsaicin exacerbated the gut microbiota imbalance in DM mice.

Indicators		Group		
		CON (n=6)	DM (n=6)	DM+CAP (n=6)
Unweighted unifrac	Mean	0.3402	0.4019	0.4309
	SD	0.02630	0.03012	0.03939
	SEM	0.006790	0.007778	0.01017
	Lower 95% CI	0.3256	0.3852	0.4091
	Upper 95% CI	0.3548	0.4186	0.4527
Chao1 index	Mean	674.3	606.1	560.3
	SD	37.47	54.39	49.62
	SEM	15.30	22.21	20.26
	Lower 95% CI	634.9	549.0	508.2
	Upper 95% CI	713.6	663.2	612.4
ACE index	Mean	671.4	618.3	558.8
	SD	36.70	61.79	53.27
	SEM	14.98	25.23	21.75
	Lower 95% CI	632.9	553.4	502.9
	Upper 95% CI	709.9	683.1	614.7

	Mean	0.09097	0.09419	0.08432
	SD	0.04550	0.03194	0.04571
Simpson index	SEM	0.01857	0.01304	0.01866
	Lower 95% CI	0.04322	0.06067	0.03635
	Upper 95% CI	0.1387	0.1277	0.1323
	Mean	3.560	3.579	3.654
	SD	0.3775	0.2736	0.4105
Shannon index	SEM	0.1541	0.1117	0.1676
	Lower 95% CI	3.163	3.292	3.223
	Upper 95% CI	3.956	3.866	4.085
	Mean	0.006539	0.000	0.0003648
	SD	0.006579	0.000	0.0008937
Akkermansia	SEM	0.002686	0.000	0.0003648
	Lower 95% CI	-0.0003655	0.000	-0.0005730
	Upper 95% CI	0.01344	0.000	0.001303
	Mean	0.06746	0.03810	0.01796
	SD	0.04615	0.02254	0.006819
Streptococcus	SEM	0.01884	0.009203	0.002784
	Lower 95% CI	0.01903	0.01444	0.01081
	Upper 95% CI	0.1159	0.06175	0.02512
	Mean	0.01475	0.05794	0.01223
	SD	0.009695	0.04732	0.009764
Faecalicoccus	SEM	0.003958	0.01932	0.003986
	Lower 95% CI	0.004579	0.008285	0.001982
	Upper 95% CI	0.02493	0.1076	0.02248
	Mean	0.05418	0.4868	0.2812
	SD	0.03183	0.2573	0.3360
Alistipes	SEM	0.01299	0.1051	0.1372
	Lower 95% CI	0.02078	0.2167	-0.07137
	Upper 95% CI	0.08758	0.7569	0.6338
	Mean	0.06931	0.2266	0.3416
	SD	0.08868	0.1423	0.4039
Anaerotruncus	SEM	0.03620	0.05810	0.1649
	Lower 95% CI	-0.02375	0.07727	-0.08232
	Upper 95% CI	0.1624	0.3760	0.7655

Acetatifactor muris	Mean	0.04154	0.4131	0.2429
	SD	0.04959	0.4092	0.06997
	SEM	0.02025	0.1670	0.02856
	Lower 95% CI	-0.01051	-0.01625	0.1694
	Upper 95% CI	0.09358	0.8425	0.3163
Allobaculum	Mean	2.893	0.009165	0.002185
	SD	1.687	0.01261	0.001967
	SEM	0.6888	0.005149	0.0008031
	Lower 95% CI	1.123	-0.004071	0.0001203
	Upper 95% CI	4.664	0.02240	0.004249
Olsenella	Mean	0.08778	0.0003115	0.000
	SD	0.07431	0.0007630	0.000
	SEM	0.03033	0.0003115	0.000
	Lower 95% CI	0.009804	-0.0004892	0.000
	Upper 95% CI	0.1658	0.001112	0.000
Erysipelotrich-aceae	Mean	2.989	0.5672	0.05497
	SD	1.729	0.8130	0.05332
	SEM	0.7060	0.3319	0.02177
	Lower 95% CI	1.174	-0.2860	-0.0009811
	Upper 95% CI	4.804	1.420	0.1109
Barnesiella intestinihominis	Mean	11.71	5.177	0.000
	SD	4.870	3.247	0.000
	SEM	1.988	1.325	0.000
	Lower 95% CI	6.601	1.770	0.000
	Upper 95% CI	16.82	8.584	0.000
Eubacterium uniforme	Mean	0.1264	0.1842	0.01411
	SD	0.1023	0.2054	0.02350
	SEM	0.04175	0.08386	0.009592
	Lower 95% CI	0.01903	-0.03135	-0.01055
	Upper 95% CI	0.2337	0.3998	0.03877

Table S5. Serum metabolomics detected by untargeted metabolomics.

Indicators		Group CON (n=6)	Group DM (n=6)	Group DM+CAP (n=6)
Dopamine	Mean	6353027	2259877	2250267
	SD	2896828	814780	891147
	SEM	1182625	332633	363809
	Lower 95% CI	3312992	1404818	1315066
	Upper 95% CI	9393061	3114936	3185468
Epinephrine	Mean	6452362	4456827	6997926
	SD	1280936	1209733	4383304
	SEM	522940	493871	1789476
	Lower 95% CI	5108103	3187291	2397930
	Upper 95% CI	7796622	5726364	11597922
Acetylcholine	Mean	21400000	14166473	16600000
	SD	1525778	3892688	5502727
	SEM	622896	1589183	2246479
	Lower 95% CI	19798794	10081347	10825243
	Upper 95% CI	23001206	18251599	22374757
Cortisol	Mean	1599104	12839009	13700000
	SD	979506	8515049	3027871
	SEM	399882	3476254	1236123
	Lower 95% CI	571176	3903013	10522445
	Upper 95% CI	2627033	21775005	16877555
Butyric acid	Mean	406833333	222333333	176500000
	SD	137870108	93762821	45196239
	SEM	56285236	38278512	18451287
	Lower 95% CI	262147528	123935287	129069456
	Upper 95% CI	551519138	320731380	223930544

L-Tryptophan	Mean	132833333	95066667	83083333
	SD	23811062	31168296	28099496
	SEM	9720825	12724404	11471571
	Lower 95% CI	107845156	62357546	53594721
	Upper 95% CI	157821510	127775787	112571946
5-Hydroxy-L-tryptophan	Mean	12183333	8493845	7738520
	SD	1070358	2079992	2279787
	SEM	436972	849153	930719
	Lower 95% CI	11060061	6311027	5346031
	Upper 95% CI	13306605	10676663	10131010
5-hydroxytryptamine	Mean	6141856	4254150	3435915
	SD	1534219	1028762	1162047
	SEM	626342	419990	474404
	Lower 95% CI	4531792	3174530	2216421
	Upper 95% CI	7751921	5333770	4655409
5-Hydroxyindole-acetate	Mean	15350000	8818618	8324198
	SD	2512966	2087884	2368986
	SEM	1025914	852375	967135
	Lower 95% CI	12712804	6627517	5838099
	Upper 95% CI	17987196	11009718	10810297
Xanthurenic acid	Mean	8561798	5153590	16094914
	SD	3405577	2339757	6584608
	SEM	1390321	955202	2688155
	Lower 95% CI	4987864	2698165	9184792
	Upper 95% CI	12135732	7609014	23005036
Indole-3-pyruvic acid	Mean	6770336	3030743	3018657
	SD	2939044	1837811	3271058

	SEM	1199860	750283	1335404
	Lower 95% CI	3685999	1102079	-414108
	Upper 95% CI	9854674	4959407	6451422
Indole	Mean	27816667	20366667	18816667
	SD	2699938	6730726	5459457
	SEM	1102245	2747807	2228814
	Lower 95% CI	24983255	13303203	13087318
	Upper 95% CI	30650078	27430130	24546015
Indole-3-acetic acid	Mean	63050000	54383333	110900000
	SD	9357510	9708639	29835214
	SEM	3820188	3963535	12180175
	Lower 95% CI	53229895	44194742	79589863
	Upper 95% CI	72870105	64571925	142210137
Hippurate	Mean	319666667	969333333	472333333
	SD	49399055	263543292	412283236
	SEM	20167080	107591098	168313926
	Lower 95% CI	267825537	692761610	39668612
	Upper 95% CI	371507796	1245905056	904998055
N-Acetyl-L-phenylalanine	Mean	39733333	67783333	48416667
	SD	7004475	14409499	10127273
	SEM	2859565	5882653	4134442
	Lower 95% CI	32382588	52661491	37788745
	Upper 95% CI	47084079	82905175	59044588

Table S6. Effect of dietary capsaicin on the expression of intestinal tight junction protein ZO-1 and Occludin in DM mice.

Indicators		Group CON (n=3)	Group DM (n=3)	Group DM+CAP (n=3)
ZO-1	Mean	2.304	1.253	0.4182
	SD	0.3205	0.2633	0.09547
	SEM	0.1850	0.1520	0.05512
	Lower 95% CI	1.508	0.5987	0.1810
	Upper 95% CI	3.100	1.907	0.6553
Occludin	Mean	2.642	2.236	1.154
	SD	0.5876	0.1505	0.4543
	SEM	0.3392	0.08687	0.2623
	Lower 95% CI	1.182	1.862	0.02555
	Upper 95% CI	4.101	2.610	2.283
	Upper 95% CI	47084079	82905175	59044588

Table S7. Gut microbiota depletion exacerbates anxiety and depressive-like behaviors in DM mice with capsaicin diet.

Indicators		Group		
		CON (n=3)	DM (n=3)	DM+CAP (n=3)
Distance in center of OFT in ABX experiment	Mean	69.56	44.49	21.21
	SEM	2.262	2.129	2.071
		Group		
		CON+ABX (n=3)	DM+ABX (n=3)	DM+CAP+ABX (n=3)
	Mean	74.45	6.083	3.463
	SEM	9.699	10.43	3.313
		Group		
		CON (n=3)	DM (n=3)	DM+CAP (n=3)
Time in center of OFT in ABX experiment	Mean	11.08	4.063	3.457
	SEM	1.023	1.969	1.073
		Group		
		CON+ABX (n=3)	DM+ABX (n=3)	DM+CAP+ABX (n=3)
	Mean	10.06	0.1867	0.6467
	SEM	4.844	1.969	1.073

		Group		
		CON (n=3)	DM (n=3)	DM+CAP (n=3)
Traveled distance of OFT in ABX experiment	Mean	419.9	241.9	153.0
	SEM	50.92	7.725	8.401
		Group		
		CON+ABX (n=3)	DM+ABX (n=3)	DM+CAP+ABX (n=3)
	Mean	442.4	45.03	60.70
	SEM	2.485	3.006	3.995
		Group		
		CON (n=3)	DM (n=3)	DM+CAP (n=3)
Immobility time in FST	Mean	176.1	175.9	191.9
	SEM	5.593	9.159	1.764
		Group		
		CON+ABX (n=3)	DM+ABX (n=3)	DM+CAP+ABX (n=3)
	Mean	173.2	226.1	225.6
	SEM	3.743	2.838	2.682

Statistical chart indicators include the group and P-values.

Indicators		Comparison group	
		DM vs CON	DM+CAP vs DM
Time in center in OFT	P-values	0.0442	0.0066
Entries in center in OFT	P-values	0.0404	0.0031
Distance in center in OFT	P-values	0.0183	0.0105
Distance in open arms in EPM	P-values	0.9437	<0.0001
Time in open arms in EPM	P-values	0.9986	0.0141
Total disatance in EPM	P-values	>0.9999	0.0440

Total distance in OFT	P-values	0.0005	0.0003
Immobility time in TST	P-values	0.0183	0.0251
Immobility time in FST	P-values	0.4976	0.0041
MWM Day0 Latency to platform	P-values	0.5688	0.9151

Indicators		Comparison group	
		DM vs CON	DM+CAP vs DM
Metanephrine	P-values	0.0099	>0.9999
Tyramine	P-values	0.0463	0.1487
Glycine	P-values	0.0237	0.5980
Epinephrine	P-values	0.0041	0.9146
L-histidine	P-values	0.0443	0.1000
L-glutamine	P-values	0.0007	0.6628
Acetylcholine	P-values	0.0464	0.3512
Kynurenic acid	P-values	0.0593	0.0356
Succinic acid	P-values	0.6026	0.0343

Indicators		Comparison group	
		DM vs CON	DM+CAP vs DM
Unweighted unifrac	P-values	0.0007	0.0344
Chao1 index	P-values	0.0320	0.0384
ACE index	P-values	0.3887	0.0195
Simpson index	P-values	>0.9999	>0.9999

Shannon index	P-values	0.9262	0.9231
Akkermansia	P-values	0.0196	0.8713
Streptococcus	P-values	0.1883	0.0196
Faecalicoccus	P-values	0.0577	0.0277
Alistipes	P-values	0.0159	0.1667
Anaerotruncus	P-values	0.0467	0.5067
Acetatifactor muris	P-values	0.0349	0.2406
Allobaculum	P-values	0.0002	0.9999
Olsenella	P-values	0.0060	0.9901
Erysipelotrichaceae	P-values	0.0035	0.4340
Barnesiella intestinihominis	P-values	0.0143	0.0479
Eubacterium uniforme	P-values	0.4634	0.0839

Indicators		Comparison group	
		DM vs CON	DM+CAP vs DM
Dopamine	P-values	0.0028	0.9928
Epinephrine	P-values	0.0386	0.2386
Acetylcholine	P-values	0.0134	0.3075
Cortisol	P-values	0.0042	0.7802
Butyric acid	P-values	0.0118	0.4385
L-Tryptophan	P-values	0.0349	0.4677
5-Hydroxy-L-tryptophan	P-values	0.0081	0.4985

5-hydroxytryptamine	P-values	0.0402	0.2783
5-Hydroxyindoleacetate	P-values	0.0004	0.7184
Xanthurenic acid	P-values	0.2082	0.0015
Indole-3-pyruvic acid	P-values	0.0442	0.9940
Indole	P-values	0.0421	0.6159
Indole-3-acetic acid	P-values	0.4395	0.0002
Hippurate	P-values	0.0025	0.0084
N-Acetyl-L-phenylalanine	P-values	0.0010	0.0079

Indicators		Comparison group	
		DM vs CON	DM+CAP vs DM
ZO-1	P-values	0.0035	0.0106
Occludin	P-values	0.4634	0.0405

Indicators		Comparison group		
		CON vs CON+ABX	DM vs DM+ABX	DM+CAP vs DM+CAP+ABX
Distance in center of OFT in ABX experiment	P-values	0.9225	0.0020	0.0123
Time in center of OFT in ABX experiment	P-values	0.8509	0.0186	0.0491
Traveled distance of OFT in ABX experiment	P-values	0.6944	0.0001	0.0003
Immobility time in FST	P-values	0.6332	0.0138	0.0006
